# Effect of skull contours on dose calculations in Gamma Knife Perfexion stereotactic radiosurgery

**DOI:** 10.1120/jacmp.v15i2.4603

**Published:** 2014-03-06

**Authors:** Hisato Nakazawa, Masataka Komori, Yoshimasa Mori, Masahiro Hagiwara, Yuta Shibamoto, Takahiko Tsugawa, Chisa Hashizume, Tatsuya Kobayashi

**Affiliations:** ^1^ Department of Radiological Sciences Nagoya University Graduate School of Medicine Nagoya Aichi Japan; ^2^ Nagoya Radiosurgery Center Nagoya Kyoritsu Hospital Nagoya Aichi Japan; ^3^ Department of Radiology Nagoya City University Graduate School of Medical Sciences Nagoya Aichi Japan

**Keywords:** skull measurements, outer contouring, dose calculation, stereotactic radiosurgery, Gamma Knife

## Abstract

In treatment planning of Leksell Gamma Knife (LGK) radiosurgery, the skull geometry defined by generally dedicated scalar measurement has a crucial effect on dose calculation. The LGK Perfexion (PFX) unit is equipped with a cone‐shaped collimator divided into eight sectors, and its configuration is entirely different from previous model C. Beam delivery on the PFX is made by a combination of eight sectors, but it is also mechanically available from one sector with the remaining seven blocked. Hence the treatment time using one sector is more likely to be affected by discrepancies in the skull shape than that of all sectors. In addition, the latest version (Ver. 10.1.1) of the treatment planning system Leksell GammaPlan (LGP) includes a new function to directly generate head surface contouring from computed tomography (CT) images in conjunction with the Leksell skull frame. This paper evaluates change of treatment time induced by different skull models. A simple simulation using a uniform skull radius of 80 mm and anthropomorphic phantom was implemented in LGP to find the trend between dose and skull measuring error. To evaluate the clinical effect, we performed an interobserver comparison of ruler measurement for 41 patients, and compared instrumental and CT‐based contours for 23 patients. In the phantom simulation, treatment time errors were less than 2% when the difference was within 3 mm. In the clinical cases, the variability of treatment time induced by the differences in interobserver measurements was less than 0. 91%, on average. Additionally the difference between measured and CT‐based contours was good, with a difference of −0.16%±0.66% (mean ±1 standard deviation) on average and a maximum of 3.4%. Although the skull model created from CT images reduced the dosimetric uncertainty caused by different measurers, these results showed that even manual skull measurement could reproduce the skull shape close to that of a patient's head within an acceptable range.

PACS number: 87.53.Ly

## I. INTRODUCTION

**Figure 1:**
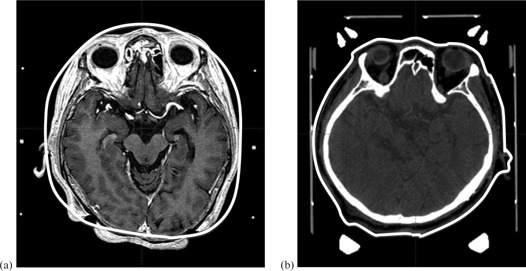
Illustrative cases of superimposed magnetic resonance imaging (a) measured using dedicated ruler and transparent hemispherical helmet, and computed tomography (CT) (b) on skull model (solid white line) created from CT image. The nose, ear, and occipital regions show large discrepancies between the skull model and actual patient's head contour (a). The skull model is completely consistent with the patient contour.

The Leksell Gamma Knife (LGK) stereotactic radiosurgery (SRS) system with a large number of radiation beams — exceeding at least a few hundred — is one of the ultimate treatment options for intracranial targets, to diverge all beams at a focus and diversify the exposure to beams for surrounding normal brain.^1^, ^2^ In the planning of LGK, treatment time (i.e., the beam‐on time to deliver the prescribed dose to the target) is calculated by composite of multiple beams emitted from the ${\;}^{60}\text{Co}$ source and by exponential attenuation allowed for the transit distance within the skull shape.^(3)^ Because the skull shape is usually provided by manual measurement based on limited measuring points using a skull measurement sphere and a dedicated ruler that measures the distance to the scalp, it differs from the actual patient contour (Fig. 1(a)). The change of treatment time caused by different skull models was previously evaluated for LGK model C,^(4)^ but this impact with the latest model LGK Perfexion (PFX) (Elekta, Stockholm, Sweden) has not been investigated. The PFX is equipped with a cone‐shaped collimator divided into eight sectors and its configuration is entirely different from that of model C. Beam delivery on the PFX is made by a combination of eight sectors, but it is also mechanically available from one sector with the remaining seven blocked. The treatment time using a single sector is more likely to be affected by discrepancies between the skull shape and actual head contour than all sectors. In addition, a computer tomography (CT)‐based skull contour function has recently been implemented in Leksell GammaPlan (LGP) Version 10.1.1 for GK treatment planning. This function allows drawing along the outline of the patient's head without effect of artifacts induced by the metallic stereotactic skull frame, posts, and fixation screws (Fig. 1(b)), and reduces differences in skull model caused by different measurers. The aims of the current work were to investigate the impact induced by expansion of the skull model on treatment time in phantom simulations, and in clinical cases, the change of treatment time by differences of skull delineation produced by manual scaling compared with CT‐based scaling and by variations in intermeasurer scale reading in PFX treatment.

## II. MATERIALS AND METHODS

### A. Source and collimator configuration of LGK Perfexion

It is useful here to describe the characteristics of the PFX collimator. An array of 192 cobalt‐60 sources is arranged in a cone‐shaped configuration. Only three types of collimator aperture sizes are available (4, 8, and 16 mm); this is different from models B and C which had four collimator sizes (4, 8, 14, and 18 mm). The collimator ring is subdivided into eight identical sectors, each sector containing 24 sources and 72 collimators (24 collimators for each of the three collimator sizes). Conformal and invaginated dose distributions are produced by using multiple shots, in combination with sector combinations.

### B. Clinical workflow of LGK stereotactic radiosurgery

The procedure on the day of LGK treatment begins with Leksell skull frame placement on the patient's head under injection of local anesthetics. After the frame fixation, contrast‐enhanced magnetic resonance imaging (MRI) and CT are acquired with the frame. These images loaded in LGP are registered with the stereotactic coordinate system using the fiducial markers on the localizer box attached to the frame. Radiosurgical dosimetry planning for the target is performed to achieve appropriate dose and dose distribution by using single or multiple shots, adjusting the each shot weight, and selecting the collimator size. A detailed description of LGK treatment workflow has already been reported.^5^, ^6^

### C. Calculation of treatment time on LGP

**Figure 2:**
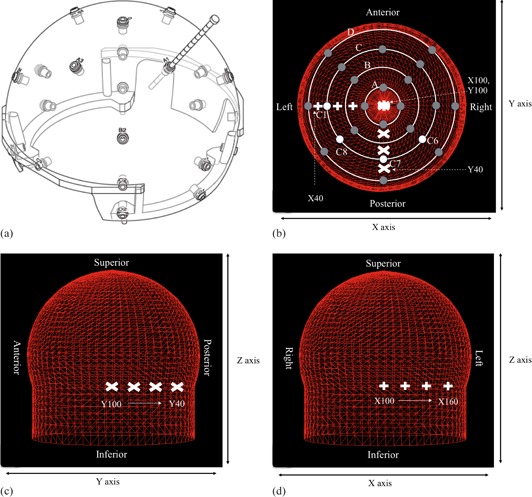
Hemispherical skull measurement helmet and series of single shot treatment planning used to investigate the change of shot position on skull model discrepancies, showing helmet and ruler (a). Top view (b) of the 24 measurement points in the helmet (dark grey circles; white circles are measurement points C1, C6, C7, and C8); lateral view (c) and frontal view (d) of skull model with all measurement points set to 80 mm. The shot position of y‐axis (c) and x‐axis (d) is shown in white mark.

Treatment time is determined by the prescribed dose for the target, dose rate of the ${\;}^{60}\text{Co}$ source, collimator size including selection of different collimator sizes for each sector, and the transit distance of the beam for the skull shape, and is calculated using the simple tissue‐maximum ratio (TMR) 10 method employing the measurement‐based dose calculation by replacing all anatomical structures with water‐equivalent material.^7^, ^8^ TMR 10 dose algorithm is available in LGP ver. 10 and later. Here, we defined treatment time as beam‐on time when a single shot is used for the target, and total treatment time as a sum of the beam‐on times for each shot when multiple shots are used. Additionally, in case of multiple targets, prescribed dose was determined for each target. In the multishot planning for the specific target, prescribed dose was provided for specific isodose of dose distribution made from combination of the shots. Hence, treatment time of each shot was calculated according to relative weight of the individual shot for the prescribed dose. Two methods are used for obtaining the patient skull model in LGP. One is created by measuring the scale values from 24 circular holes using a transparent hemispherical skull measurement helmet and a dedicated ruler that measures the distance to the scalp (Fig. 2(a)). The 24 measuring points consist of the patient's top radius and 23 other positions arranged in eight longitudinal columns, with lateral rings designed A, B, C, and D (Fig. 2(b)). The other method is CT‐based skull modeling and this requires acquisition of the whole of the skull, including the frame. A CT image with a resolution of 512×512 pixels in the axial plane and a slice thickness of 1.25 mm is adopted to reduce partial volume effects.

### D. Simulation of discrepancy using phantoms

**Figure 3:**
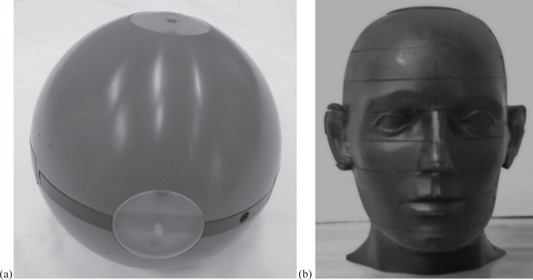
Spherical polystyrene phantom (a) of diameter 160 mm provided by vender (Elekta); anthropomorphic head phantom simulated human head structures (b).

A spherical polystyrene phantom 160 mm in diameter provided by the vendor (Elekta, Stockholm, Sweden) for dose calibration of PFX (Fig. 3(a)) and an anthropomorphic phantom which simulates human head structures (Fig. 3(b)) were used to examine the effect induced by discrepancies of skull measurements on treatment time. The phantom simulation was performed in an experimentally simple setting that employs single shot and expansion of skull contour for specific location. The collimator setting for the shot was determined for both one sector and all sectors. One sector directly reflected relationship between the discrepancies and treatment time, excluding irradiation from the other seven sectors. In LGP, the amounts of expanded skull geometry were established as 1, 2, 3, 5, 10, 15, and 20 mm from original position at the specific measuring points of the transparent hemispherical skull measurement helmet. In the spherical phantom, similar to Berndt and Beck,^(4)^ we examined the treatment time dependence on location difference for some measurement coordinates using a single shot with the 8 mm collimator from only one sector and from all sectors. The shot coordinates ranged from (x,y,z)=(100 mm,100 mm,125 mm) to (100 mm,40 mm,125 mm), incremented by Δy=20 mm (Figs. 2(b), 2(c)). The origin of Leksell coordinate system (the point where x, y, and z are numerically zero) is located outside the coordinate frame at a point that is superior, lateral, and posterior to the coordinate frame on the patient's right side. The orientations of the axes in the stereotactic space coordinates system were: × as right‐left direction, y as posterior‐anterior direction, and z as superior‐inferior direction. In the anthropomorphic phantom, the orientations of the axes were: the left ear (right ear) and rear of the head which easily generate apparent differences between observers and were compatible with C1 (C5) and C7 of the helmet, respectively. Therefore for regions close to these objects, a single shot with the 8 mm collimator was delivered from only one sector or from all sectors. The shot coordinates for expansion of skull geometry in C1 ranged from (x,y,z)=(100 mm,100 mm,125 mm) to (160 mm,100 mm,125 mm), incremented by Δx=20 mm (Figs. 2(b), 2(c)). On the other hand, the shot coordinates for expansion of skull geometry in C7 ranged from (x,y,z)=(100 mm,40 mm,125 mm) to (100 mm,40 mm,125 mm), incremented by Δy=20 mm (Figs. 2(b), 2(d)). The treatment time was calculated and compared between the reference settings obtained by ruler measurement and the above amounts of expanded skull geometry from those settings.

### E. Variation of treatment time caused by measurement uncertainty in clinical cases

A total of 64 patients were enrolled in a clinical study. All patients underwent LGK SRS between January 2011 and February 2013. Variations of treatment time for each shot and total treatment time between observers were recorded in 41 patients with single or multiple metastatic brain tumors on LGP. The skull measurements were conducted by two radiation technologists with much experience in LGK SRS. The treatment time between the two was evaluated as the average, maximum, and minimum difference (%). A comparison of the total treatment time and time for each shot by skull model between CT‐based contour and manual skull measurement was performed for 23 patients with acoustic neurinoma. In addition, the same 23 patients were evaluated for the change of treatment time for various locations within treatment range in LGK between CT‐based contour and manual skull measurement. A single shot with the 8 mm collimator from all sectors was placed in different locations from the epipharynx to the top of the head (epipharynx, cerebellar, inter canal, pons, temporal, optic nerve, eye, third cerebroventricle, frontal, parietal, and parietal bone) for LGK SRS. Because a shot from one sector is not used so often in clinical cases, its impact was not investigated. On LGP, the setting for the automatic CT contour was 1.0 mm of grid size and the window width of CT numbers ranged from −1500 to 500. Collected data of the treatment time were analyzed using SPSS version 18.0 (IBM, Japan). The paired *t*‐test was used to examine differences in treatment time between CT‐based contour and manual skull measurements. Differences with p<0.05 were regarded as significant.

## III. RESULTS

### A. Simulation of discrepancy using phantoms

**Figure 4:**
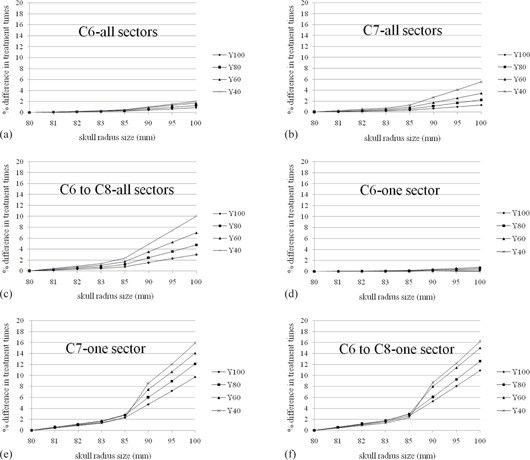
Percentage difference in treatment times for single shot treatment from all sectors ((a), (b), (c)) or one sector ((d), (e), (f)) generated with the default setting of the 80 mm radius hemispherical skull model and for the same treatment with discrepancies (1 mm, 2 mm, 3 mm, 5 mm, 10 mm, 15 mm, 20 mm) from 80 mm. C6, C7, and C8 are measurement point of a hemispherical skull measurement helmet and were expanded on above discrepancies. The measurement coordinates ranged from (x,y,z)=(100 mm,100 mm,125 mm) to (100 mm, 40 mm, 125 mm), incremented by Δy=20 mm. The difference of treatment time increases with increases in skull radius size and distance from center location. One sector was affected significantly larger than all sectors.

**Figure 5:**
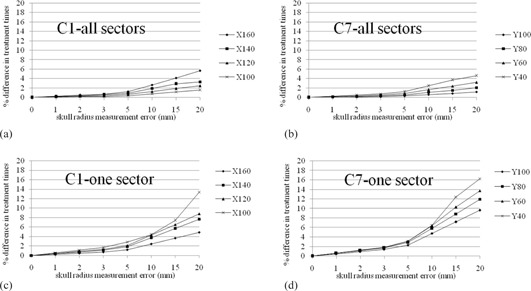
Percentage difference in treatment times for single shot treatment from all sectors ((a), (b)) or one sector ((c), (d)) in the anthropomorphic phantom and for the same treatment with discrepancies (1 mm, 2 mm, 3 mm, 5 mm, 10 mm, 15 mm, 20 mm) from the original skull model. C1 and C7 are measurement point of a hemispherical skull measurement helmet and were expanded on above discrepancies from original values. The measurement coordinates for C1 ranged from (x,y,z)=(100 mm,100 mm,125 mm) to (160 mm, 100 mm, 125 mm), incremented by Δx=20 mm. The measurement coordinates for C7 ranged from (x,y,z)=(100 mm,100 mm,125 mm) to (100 mm, 40 mm, 125 mm), incremented by Δy=20 mm. The difference of treatment time increases with increases in skull radius size and distance from center location. One sector was affected significantly larger than all sectors.

For the hemispherical skull model, Fig. 4 shows the differences in treatment time for all sectors and one sector corresponding to intentionally measuring errors from 1 to 20 mm. The percentage differences in treatment times for the anthropomorphic phantom between a reference setting and the discrepancy from it are presented in Fig. 5.

### B. Variation of treatment time caused by measurement uncertainty in clinical cases

Table 1 summarizes the discrepancies between the corresponding treatment times for the measurements of two observers in 41 patients with brain metastases. In Table 2, we compare the treatment times of CT‐based delineation and ruler measurements for 23 patients with acoustic neurinoma. There was no significant difference between CT and manual measurement techniques (p=0.61). In addition, Table 3 demonstrates the impact of intracranial various lesions on treatment time errors for the cases listed in Table 2.

**Table 1:** Differences in treatment time between two measurers for 41 patients with brain metastases

*Case Number*	*Shot Number*	*Treatment Time A (min.)*	*Treatment Time B (min.)*	*Mean Difference (%)*	*Max. Difference (%)*	*Min. Different (%)*
1	37	150.89	148.5	−1.58	−2.1	−1.23
2	18	121.75	120.48	−1.04	−1.63	−0.52
3	12	54.64	52.15	−4.56	−4.81	−4.32
4	2	17.31	17.04	−1.56	−1.65	−1.47
5	5	49.73	49.03	−1.41	−1.46	−1.35
6	7	46.88	46.1	−1.66	−1.91	−1.17
7	15	32.19	31.23	−2.98	−4.07	−2.05
8	6	69.32	68.57	−1.08	−1.83	−0.77
9	12	54.34	53.71	−1.16	−1.51	−0.77
10	1	13.37	13.18	−1.42		
11	8	30.52	29.93	−1.93	−6.11	−0.68
12	10	88.53	86.82	−1.93	−2.95	−0.4
13	14	97.47	96.21	−1.29	−2.25	−0.69
14	19	150.64	148.09	−1.69	−2.39	−0.4
15	13	101.79	100.18	−1.58	−2.11	−0.88
16	23	112.77	111.19	−1.40	−2.1	−0.81
17	17	72.34	71.43	−1.26	−1.79	−0.88
18	17	66.11	64.85	−1.91	−3.03	−0.86
19	6	26.63	26.32	−1.16	−1.44	−1.06
20	22	144.16	143.7	−0.32	4.17	0.12
21	9	31.4	31.02	−1.21	−1.65	−0.77
22	15	129.06	128.26	−0.62	−1.36	−0.12
23	10	50.16	49.78	−0.76	−0.87	−0.36
24	1	8.17	8.06	−1.35		
25	7	20.06	19.64	−2.09	−2.25	−1.52
26	36	146.02	146.4	0.26	1.54	0
27	42	135.72	134.13	−1.17	−1.73	0
28	41	216.63	214.33	−1.06	−4.48	0
29	1	12.84	12.7	−1.09		
30	6	26.09	25.8	−1.11	−1.25	−1.01
31	3	18.25	18.04	−1.15	−1.34	−0.99
32	2	19.33	19.13	−1.03	−1.29	−0.83
33	4	22.14	21.79	−1.58	−1.6	−1.53
34	25	87.03	86.56	−0.54	−1.1	0
35	1	18.52	18.38	−0.76		
36	16	92.14	91.45	−0.75	−1.54	0.12
37	6	41.69	41.54	−0.36	−0.51	−0.24
38	18	81.87	80.77	−1.34	−1.87	−0.88
39	26	87.61	87.02	−0.67	−0.96	−0.44
40	3	25.99	25.9	−0.35	−0.43	−0.22
41	8	51.47	51.3	−0.33	−0.69	0
Average	13	68.87	68.07	−1.16	−1.74	−0.78
S.D	11	50.10	49.64	−0.91	1.66	0.80

**Table 2:** Percentage difference of treatment time between CT‐based and measured skull shape for 23 patients with acoustic neurinoma

*Case Number*	*Shot Number*	*Treatment Time (min.) CT‐base*	*Treatment Time (min.) Measurement*	*Difference* (%)
1	20	45.88	45.33	−1.20
2	7	29.8	30.07	0.91
3	20	51.42	51.86	0.84
4	23	45.76	44.23	−3.34
5	12	36.66	35.4	−3.44
6	11	29.9	30.05	0.50
7	5	19.51	19.35	−0.82
8	24	74.5	75.12	0.83
9	36	62.8	61.69	−1.77
10	22	55.6	56.3	1.26
11	20	55	54.31	−1.25
12	15	44.8	44.74	−0.13
13	20	51.85	51.93	0.15
14	22	61.92	62.42	0.81
15	23	46.9	47.26	0.77
16	18	42.39	43.78	3.28
17	21	56.83	56.98	0.26
18	23	56.62	56.42	−0.35
19	7	24.46	24.4	−0.25
20	14	38.93	38.79	−0.36
21	14	30.36	30.58	0.72
22	6	27.08	26.63	−1.66
23	9	31.74	31.4	−1.07
Average	17	44.38	44.31	−0.16
S.D	7	14.14	14.24	0.66

**Table 3:** Percentage difference of treatment time in single 8 mm shot virtually placed in various intracranial lesions between CT‐based and measured skull shape

*Isocenter Location*	*Average (%)*	*S.D. (%)*
Right epipharynx	−0.87	1.09
Left epipharynx	−0.70	1.08
Right cbll	−4.54	3.12
Left cbll	−4.26	3.18
Contradiction intercanal	−0.86	1.08
Pons	−1.18	1.33
Right temporal	0.08	0.95
Left temporal	0.16	0.71
Optic nerve	−0.62	1.07
Right eye	−0.60	1.24
Left eye	−0.83	1.12
Third cerebroventricle	−0.37	0.62
Right frontal	0.92	0.80
Left frontal	0.79	0.89
Right parietal	−0.25	1.17
Left parietal	−0.29	1.09
Parietal bone	0.10	2.43

## IV. DISCUSSION

In this work, we evaluated the effect of the different skull model on treatment time both in a phantom and in a clinical study. Two kinds of phantoms were used for the simulation study. The spherical phantom evaluated the basic features of treatment time resulting from skull contour discrepancies, while the anthropomorphic phantom was used to make the simulation more relevant to the clinical setting. Skull models with discrepancies of less than 3 mm caused treatment time differences of 2% or less. The treatment time errors resulting from skull model discrepancies of 20 mm were 10% and 16% for full 192 beams using 8 sectors and 24 beams using only 1 sector, respectively (4, 5). The trend obtained from those results was similar to the report of Berndt and Beck,^(4)^ in that centrally located shots were less affected by overestimation in skull contour; however, when the shots moved toward the discrepancy and the beam weight passing through inaccurate area of skull contour increased (namely, use of single sector), the treatment time error significantly increased according to the degree of discrepancy. We have to pay special attention when a single sector is delivered in PFX.

In the 41 clinical case studies, the interobserver total treatment time differences were 0.91%, on average. Almost cases agreed well, within 2%, but a maximum treatment time difference of 6.11% was observed for one of the 8 shots in case 11. In this case, the C1 (right ear) and D5 (left ear) of the helmet compatible with the ear area caused the largest discrepancies. The total treatment time by comparison between CT‐based and measured skull shape agreed well with −0.16%±0.66% (mean ±1 standard deviation), on average, for the 23 acoustic neurinoma cases, and showed a maximum of 3.44% for case 5. These results showed relatively good agreement between the two techniques, and demonstrated that it is possible to make an accurate patient contour with only a limited number points, although Berndt and Beck^(4)^ suggested that a large number of model points are required.

In addition, multitargets were virtually set on various intracranial lesions of these patients and each target was planned using a single shot of the 8 mm collimator at a matrix size of 1.0. As a result, in almost all of the target positions, the treatment time was influenced by the difference of skull shape by 1% or less in all cases, while the discrepancies of pons and cerebellar lesions were 1.18% and 4.54%, respectively. Because the skull shape below the D ring of the hemispherical helmet is extrapolated linearly (sagittal view of Fig. 1(a)), it is more difficult to estimate the accurate dose than for other scaling points for targets located caudal to the occipital area.

The skull contour of a patient directly acts on the dose (treatment time) and dose distribution in dose calculation for treatment planning in LGK. Several skull models have been presented by previous publications,^7^, ^8^, ^9^ and the authors have investigated only the dose discrepancy induced by different skull models. We also examined the change of dose distribution in PFX, but this showed little change (data not shown) between the initial skull model of a sphere with a radius of 80 mm before definition of the skull contour and actual patient‐specific shapes, based on our past experience.

In general, skull shape is confirmed only by visual judgment during the course of treatment planning and its dosimetric impact is not considered an important factor. CT‐based skull delin‐eation can reduce the uncertainty of the skull model created by a measurer^4^, ^9^ and, therefore, the uncertainty of the prescribed dose is also small.

## V. CONCLUSIONS

The impact induced by different skull model on treatment time was small for almost all cases. However, uncertainty of skull model can be reduced by means of CT‐based delineation which is easily automated by LGP.
